# Quality improvement of undergraduate courses based on fuzzy analytic hierarchy process and entropy method

**DOI:** 10.3389/fpsyg.2022.892628

**Published:** 2022-08-05

**Authors:** Aotian Peng

**Affiliations:** School of Business, Anyang Normal University, Anyang, China

**Keywords:** undergraduate course, analytic hierarchy process, fuzzy comprehensive evaluation method, quality improvement, entropy method, quantitative assessment

## Abstract

Since the curriculum is the core carrier to improve the level of talent cultivation in colleges and universities, strengthening the reform of course teaching and improving the quality of course teaching are fundamental to the survival and development of colleges and universities, and also an important part of higher education reform. In this study, a fuzzy analytic hierarchy process (AHP) and an entropy method were used to determine the weight of the core evaluation indicators of undergraduate course quality improvement, including four first-level indicators of the curriculum concept, curriculum resources, curriculum organization, and curriculum effectiveness, and 12 s-level evaluation indicators and weights. Then, based on a case study of the first-class undergraduate course “Management” of Anyang Normal University, the way to evaluate the course by the AHP and entropy method was explained. Finally, according to the evaluation results, the ideas of course construction were put forward, such as changing the course concept, enriching the course resources, paying attention to the course organization, and ensuring the course effectiveness, so as to improve the quality of undergraduate courses and also to improve the quality of undergraduate talent training with the improvement of course quality as the starting point.

## Introduction

Not only is the new stage of social development and the new round of scientific and technological revolution reshaping the world’s innovation landscape but also the resulting industrial changes are further restructuring the world’s economic structure. In addition, the social requirements for talent cultivation in colleges and universities are increasingly diverse and more standardized, which leads to the redefinition of talent cultivation.

The connotation of university is promoted from three aspects: discipline construction, emphasizing the construction of knowledge category; major construction, emphasizing the organization of specialized knowledge systems according to the needs of personnel training (Bi, 2019); and curriculum construction, emphasizing the selection of a part of “the most valuable knowledge” from the subject knowledge to constitute the teaching content (Zhou, 2016), supporting the realization of the goal of talent cultivation (Wang, 2014). The commonness of the three is that they all have team construction, content construction, evaluation system construction, and funding support (Zhou and Ma, 2013). High-level undergraduate education is rooted in high-level major construction, which is based on high-quality courses, while high-quality courses are derived from first-class disciplines (Lin and Hong, 2019). The core of how to transform the achievements of discipline construction into the achievements of specialty construction is to transform them into courses (Zhang, 2016), that is, to be able to offer frontier courses and interdisciplinary courses in the course system, to be able to teach the latest achievements of discipline construction in the course content, to be able to translate the achievements of discipline construction into teaching materials in the teaching material construction, to compile high-level teaching materials that reflect the achievements of discipline construction, etc. Therefore, the connotation promotion construction of universities should adhere to the system holistic view and dynamic optimization view to promote the integration of disciplines, courses, and majors (Liu and Peng, 2018).

At present, talents with strong practical ability, high overall quality, and innovative spirit are especially needed. The curriculum is the core element of talent cultivation and the main places for talent cultivation, which plays a decisive role in shaping and improving students’ knowledge structure, ability, and personality. In this context, the relationship between teaching and learning is undergoing profound changes with the undergraduate education as the fundamental way of universities, the creation of golden courses and the continuous advancement of classroom revolution. High-quality undergraduate courses should, first, have the ability to cultivate people with moral integrity, and the ability to awaken consciousness mainly centering on the ideological and political theory of the course (Zhang et al., 2020); second, they should not only have the ability to be advanced, innovative, and challenging (Wu, 2018) but also consider the feasibility of ability training (Sun and Liu, 2020); third, on the premise of sticking to the fundamental task of cultivating people with moral integrity, they should not only have the ability to enable students to acquire knowledge and strengthen ability but also enable them to achieve the purpose of value guidance and character building, and pay attention to training students’ abilities, such as responsibility-taking ability, advanced learning ability, communication and cooperation ability, innovative practice ability, and lifelong development ability (Zhang et al., 2020); fourth, the courses should build an “excellent team” and adhere to the principles of diversified disciplinary backgrounds, pure teaching will, and standardized teaching and training in the selection and training of teacher teams (Yu, 2019); and fifth, courses should reform the “evaluation system.” As course quality and learning effectiveness are the most critical elements of high-quality courses, high-level courses should change from teacher-centered “teaching” to student-centered “enlightenment” (Lu, 2016), so as to promote the construction of an open, constructive, perceptive, and interactive teacher–student community, with the curriculum as the carrier by reconstructing the teacher–student relationship of equal dialog and mutual benefit (Cai et al., 2018). By increasing the ways of interaction, frequency, and depth of interaction (Li and Wu, 2021), an academic evaluation system oriented to diversity and creative learning outcomes (Shi et al., 2021) will be constructed, and students’ learning outcomes will be analyzed by using the big data method, and the course content, teaching means, and teaching organization will be improved at any time.

As the curriculum is the basic unit of the teaching system and an important carrier for universities to respond to the needs of times, the quality of the curriculum determines the quality of personnel training. However, there are still some outstanding problems in the current undergraduate course construction, which limit the improvement of the course quality and the construction requirements of first-class courses, mainly including the following:

### Course construction has not received enough attention

The ability of curriculum design and development of university teachers and even a university directly determines the level of its core competitiveness. Although the curriculum is the most important in a university, it has long been the most easily overlooked, which is reflected in the continuous existence of “emphasizing disciplines while neglecting courses” in the performance evaluation of universities, resulting in the curriculum construction often at the edge (Liu, 2014). The promotion of professional titles and performance assessment of university teachers often take up a larger proportion in scientific research, and teaching results are often only regarded as basic qualifications with lower requirements. Although there is no hard and fast pressure on college students to enter a higher school, their employment situation will be affected by a variety of factors, resulting in insufficient attention to the effectiveness of academic learning.

### Passive learning of students has not been fundamentally improved

In the new era, not only new requirements are put forward for talent cultivation in colleges and universities but also the way students acquire knowledge has changed greatly compared with the traditional way, and the learning situation is constantly changing. Courses in new environments not only assume the function of knowledge transmission but also are the “germs” of knowledge innovation (Xue, 2001), highlighting the enthusiasm, initiative, and expansibility of people, as independence of students is an important factor to ensure the learning effect. Therefore, it is not only necessary but also urgent to change from teaching-centered to learning-centered. However, due to various factors, many colleges and universities still focus on teachers’ imparting knowledge, in which teachers and textbooks are regarded as the authoritative source of knowledge, and successful teaching is widely understood as an effective explanation of textbooks (Liu et al., 2005), which is not conducive to the training of students’ initiative and does not meet the requirements of the new era for college graduates.

### Teachers are stricken by panic about expertise

The transformation of teaching from teacher-centered to student-centered means that the main body of teaching is shifted to students. In addition, the role of teachers has changed fundamentally, from a leader and a teacher to a leader, a supporter, and a participant, with the main tasks of arousing students’ awareness, stimulating students’ interest in learning, and building a good interactive cooperative relationship between teaching and learning. In such a scene, the requirements for teachers are higher and more comprehensive. They should not only have the accumulation of “academic disciplines” but also have the curriculum view of “ideological and political theories teaching in all courses,” and the application ability of “academic teaching” should not only have the knowledge reserve but also be familiar with the laws of teaching and learning (Boyer, 2004), so as to educate people in an all-round way. In this role adjustment, many university teachers are gradually showing some perplexities, such as limited vision, ideological shackles, weak knowledge, short ability, and lack of experience, which lead to “panic about expertise” in the teaching process (Jin, 2018; Choi et al., 2021; Zheng et al., 2021c).

In the face of such problems, China has also issued a series of policies in recent years in an attempt to promote the reform and construction of undergraduate courses from the edge of university work to the center under the guidance of the policies. In June 2018, the “National Conference on Undergraduate Education in Colleges and Universities in the New Era” was held, which was a conference to revitalize undergraduate education in an all-round way and was aimed at improving the quality of undergraduate education and teaching. At this conference, the concept of “golden courses” was formally proposed for the first time. Subsequently, in the documents issued by the Ministry of Education and the “China University Teaching Forum” held in November, the main leaders of the Ministry of Education further specified the requirements for the construction of “golden courses” and defined the standards of “golden courses” and “frivolous courses.” In November 2019, the Ministry of Education, at the national level, proposed to start the construction of first-class undergraduate courses and planned to implement the “double first-class majors” program for first-class undergraduate courses in the country’s undergraduate colleges in 3 years, that is, about 10,000 first-class undergraduate courses were identified at the national and provincial levels, respectively.

At present, the theoretical research studies on the improvement of curriculum quality mostly adopt the qualitative method, but they are too subjective. This study aims to combine the FAHP with the entropy method under this background, not only to evaluate the curriculum quality indicator but also to promote the quality of the curriculum, which is beneficial to the teaching results of online + offline courses and thus to ensure the quality of undergraduate talent training. First, the core indicators of course quality evaluation were selected, then the fuzzy analytic hierarchy process (FAHP) and entropy method were selected to determine the weight of the evaluation indicators by using a quantitative method, and then a specific course was quantitatively evaluated. On this basis, the specific method to improve the quality of undergraduate course was proposed.

## Methodology and modeling

### Methodology

The curriculum evaluation indicator system designed in this study has obvious hierarchical characteristics and has both quantitative and qualitative indicators, so it can be analyzed by using the FAHP and entropy method. The FAHP is used to obtain subjective weights according to expert opinions, and its analysis is logical and credible. In order to compensate for the subjectivity of judging the importance of each indicator, the entropy method is used to obtain the objective weight according to the data itself, which can modify the weight obtained by the FAHP. Finally, the combination of the two is a subjective and objective comprehensive weighting method, which improved the reliability of the indicator system. Obviously, the combined FAHP-entropy method can not only reflect the actual experience of experts but also make full use of existing data to reflect its potential law, and finally get a more accurate and reasonable evaluation result.

#### Fuzzy analytic hierarchy process

The FAHP was put forward by American strategist Sadie, aiming at solving the complex problems of multi-objective decision-making involving qualitative factors (Shen et al., 2021; Yuan and Li, 2021). It is simple and easy to understand, but at the same time, it cannot accurately measure some indicators when constructing the judgment matrix (Huang, 2021; Zheng et al., 2021a,b). Therefore, the combination of a fuzzy comprehensive evaluation method based on fuzzy mathematics and an analytic hierarchy process can reduce some disadvantages of the AHP. In the fuzzy comprehensive evaluation method, the influencing factors which are difficult to quantify and fuzzy in the research object are taken as a set, the corresponding membership functions are constructed by using the membership theory, and the evaluation results are presented in the form of fuzzy sets (Alyamani and Long, 2020; Fu et al., 2020; Xu et al., 2020; Wang et al., 2021). Therefore, in the fuzzy analytic hierarchy process (FAHP), which combines the fuzzy comprehensive evaluation method with the analytic hierarchy process, the fuzziness of multi-objective decision-making can be better considered, and the problem that the evaluation object is highly influenced by subjective factors can be solved.

The FAHP operates as follows:

Step 1: Determine the factor set. The set of factors affecting the evaluation object is called the factor set, which is generally expressed by *U*, *U* = (*U*_1_, *U*_2_, *U*_3_, …, *U_n_*).
Step 2: Build a hierarchical structure. The influencing factors mentioned in the previous steps were decomposed layer by layer to construct a bottom-up evaluation indicator hierarchical structure model.
Step 3: Establish the fuzzy judgment matrix of influencing factors at all levels. By issuing expert questionnaire, the pairwise comparison of influencing factors was conducted to determine the importance of influencing factors, and the relative importance of two different factors in the same level was expressed by using the quantitative description method, and the fuzzy complementary matrix *A* = (*a_ij_*)nxn was constructed, generally with the help of 0.1–0.9 nine-scale method, as shown in Table 1. When the elements in fuzzy matrix *A* = (*a_ij_*)nxn met *a_ij_* + *a_ji_* = 1, then matrix *A* was a fuzzy complementary matrix.
Step 4: The consistency of the fuzzy complementary judgment matrix *A* was checked and converted into the fuzzy consistency matrix *R* = (*r_ij_*)nxn. Because of the subjectivity in the judgment process, the consistency of the judgment matrix needs to be checked. The fuzzy consistency matrix R could be obtained by performing mathematical transformation according to Eqs. (1) and (2) on the fuzzy complementary matrix *A* = (*a_ij_*)nxn.
Step 5: According to parameter Eq. (3) and FAHP weight Eq. (4), rank the factors that affect the evaluation object. The weight of matrix *R* was obtained through the weight formula, and then the weights of primary and secondary indicators were multiplied step by step and summarized layer by layer to obtain the comprehensive weight to get a general ranking.
Step 6: Determine the degree of membership. The quality of curriculum construction was evaluated, and the evaluation set was established. The membership function was determined by using the maximum membership degree method. The subject was evaluated by the single factor evaluation matrix.
Step 7: Get the final evaluation results and conclusions.

(1) $$r_{i}=\overset{n}{\underset{k=1}{\sum }}a_{ik}\left(i=1,2,\cdots ,n\right)$$

(2) $$r_{ij}=\frac{r_{i}-r_{j}}{2\left(n-1\right)}+0.5$$

(3) $$a=\left(n-1\right)/2$$

(4) $$w_{i}=\frac{1}{n}-\frac{1}{2a}+\frac{1}{an}\overset{n}{\underset{k=1}{\sum }}r_{ik}\left(i=1,2,\ldots n\right)$$

**Table 1:** 0.1–0.9 nine scales and their meanings.

Judgment scale a_ij_	Meanings
0.9	Element *i* is extremely important to element *j*.
0.8	Element *i* is very important to element *j*.
0.7	Element *i* is more important to element *j*.
0.6	Element *i* is slightly important to element *j*.
0.5	Element *i* is equally important to element *j*.
0.4–0.3 0.2–0.1	Inverse comparison *a_ij_* = 1–*a_ji_*

*a_ij_* stands for the importance of *i* to *j*, and *a_ji_* stands for the importance of *j* to *i*.

#### Entropy method

The entropy method mainly uses the characteristics of entropy to judge the dispersion degree of each indicator in the system through the entropy value. The greater the entropy value, the greater the dispersion degree of the indicator in the system, so the greater its weight and the greater its impact on the evaluation of the whole system (Wang et al., 2013; Yang et al., 2022).

The specific application steps of the entropy method are as follows:

Step 1: Data standardization: In order to avoid the impact of indicator differences, the original data set was standardized by using mathematical methods, such as the range standardization method. Since there is no negative index in this study, the normalization process can be performed by using Eq. (5). At the same time, to avoid 0 value after standardization interfering with the final calculation result, the processed data were unified and shifted forward by 1.
Step 2: Determine the proportion of the *i*-th evaluator in the *j*-th indicator, and the calculation formula is shown in Eq. (6).
Step 3: Solve the information entropy *E_j_* of the *j*-th indicator, and the calculation formula is as shown in Eq. (7).
Step 4: Calculate the difference coefficient *D_j_* according to Eq. (8).
Step 5: Calculate the entropy *e_j_* of each indicator according to Eq. (9).

(5) $$X_{ij}=\frac{x_{ij}-\min\left(x_{ij}\right)}{\max\left(x_{j}\right)-\min\left(x_{j}\right)}+1,i=1,2,\cdots n,j=1,2,\cdots m$$

where *i* = the serial number of evaluators;

*j* = the serial number of evaluation indicators;

*X_ij_* = the data of the *i*-th evaluator under the *j*-th evaluation indicator after normalization;

*x_ij_* = the data of the *i*-th evaluator under the *j*-th evaluation indicator in the original data;

min(*x_ij_*)=the minimum value in the original data;

max(*x_j_*)=the maximum value of evaluation indicator in the line *j*;

min(*x_j_*)= the minimum value of the evaluation indicator in the row *j*.


(6) $$\delta_{\mathrm{ij}}=\frac{X_{ij}}{\overset{n}{\underset{i=1}{\sum }}x_{ij}},i=1,2,\cdots n,j=1,2,\cdots m$$

(7) $$E_{j}=-K\overset{n}{\underset{i=1}{\sum }}\delta_{ij}\ln\left(\delta_{ij}\right),i=1,2,\cdots n,j=1,2,\cdots m$$

where K=1/ln(m),K>0, Ln is the natural log.


(8) $$D_{j}=1-E_{j},j=1,2,\cdots m$$

(9) $$e_{j}=\frac{D_{j}}{\overset{m}{\underset{j=1}{\sum }}D_{j}},j=1,2,\cdots m$$

The FAHP and entropy method were combined to calculate the comprehensive weight, and the calculation formula is shown in Eq. (10).


(10) $$\beta_{j}=\frac{e_{j}w_{j}}{\overset{m}{\underset{j=1}{\sum }}e_{j}w_{j}},j=1,2,\cdots m$$

### Establishment of curriculum quality evaluation indicator system

**Figure 1:**
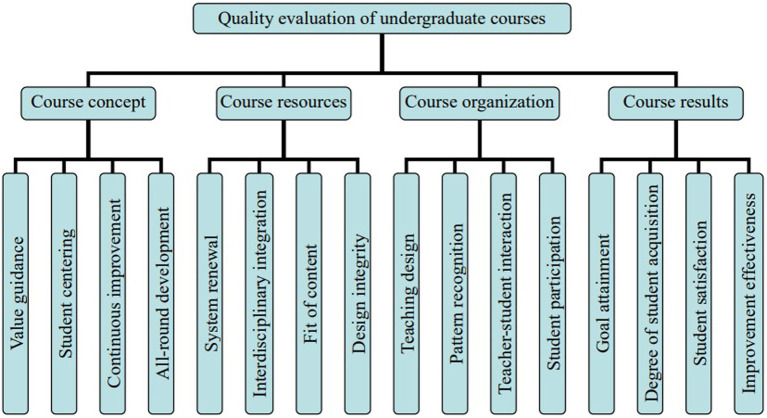
Evaluation indicator system of undergraduate courses.

Evaluating the curriculum is an effective way to improve the quality of the courses, which is to use the evaluation results of the curriculum from different perspectives by different subjects to promote the improvement of the quality of the courses. Different scholars have constructed different curriculum evaluation indicator systems. For example, Xie et al. evaluated the process of learning experience from two dimensions of emotion and cognition (Xie et al., 2021). Lei paid attention to the role of core literacy in curriculum evaluation (Lei, 2020). Wu proposed that the dialogicality should be used in the Evaluation process to improve the quality of the course under the guidance of the core literacy idea in combination with the requirements of academic quality standards (Wu, 2020). Wang designed a teaching evaluation indicator system covering the whole process of “design-implementation-effect” in the teaching process (Wang, 2021). Wang et al. designed a student-centered classroom teaching evaluation method under the OBE teaching concept, in which the students’ real experience and achievements in the learning process became the focus of attention (Wang et al., 2021). The construction of a curriculum evaluation indicator system plays a fundamental role in improving the quality of courses. Based on the study of relevant literature and the accumulated experience in the course construction, the evaluation indicator system of undergraduate courses is designed, as shown in Figure 1.

After designing the preliminary indicator of course quality evaluation, experts were invited to evaluate the indicator system, who are all teachers who teach management courses in colleges and universities with rich teaching experience and knowledge reserves. After considering all kinds of factors, ten experts were finally invited, including six women and four men, three professors, five associate professors, and two lecturers in the distribution of professional titles. After contacting the target experts and obtaining their consent to participate, information was collected by filling in the questionnaire. In the design of the questionnaire, based on the Likert scale method, each indicator was assigned a value from “1 to 10” according to the importance degree from “completely unnecessary to very necessary.” The coefficient of variation (CV), that is, the ratio of the average difference and the average value of each indicator, was used to measure the degree of coordination of the indicators. The smaller the CV is, the better the coordination of the indicators will be, that is, the members of the expert group had a high degree of consensus on this indicator. Through calculation, the CV values of each indicator finally determined in this study were all less than 0.25. Referring to the practice of others, the indicators with a score ≥ 8 and less than 80% in the questionnaire were deleted, such as the indicators of “overall development,” “design integrity,” “student participation,” and “improvement effectiveness.”

The undergraduate course quality evaluation system and the indicators at all levels are determined, as shown in Table 2, including one general target level, four first-level indicators, and 12 s-level indicators. In the evaluation system, the target level is the first-class undergraduate course quality M, and the criterion level is the four main aspects of evaluating the first-class undergraduate course quality (course concept M1, course resources M2, course organization M3, course effectiveness M4). Specific indicators at the indicator level include value guidance M11, student centering M12, continuous improvement M13, system renewal M21, fit of content M22, interdisciplinary integration M23, teaching design M31, model recognition M32, teacher–student interaction M33, goal attainment M41, student acquisition M42, and student satisfaction M43.

**Table 2:** Evaluation indicator system of undergraduate courses.

First-level indicators	Second-level indicators	Connotation of indicators
Course concept M_1_	Value guidance M_11_	Guiding the students’ emotional attitudes and values based on the principle of moral cultivation; The curriculum objectives are in line with the school orientation and personnel training objectives
	Student centering M_12_	Designing the whole teaching process based on students’ learning effectiveness
	Continuous improvement M_13_	Actively collecting data to carry out teaching reflection, teaching research and teaching improvement, and circularly optimize the curriculum
Course resources M_2_	System renewal M_21_	Content of teaching reflects the frontier of discipline and the development needs of the times.
	Fit of content M_22_	The course resources are scientific and contemporary, with various forms, and fit in with the curriculum concepts and objectives.
	Interdisciplinary integration M_23_	The course resources reflect the thinking of multidisciplinary integration, integration of disciplines and industries.
Course organization M_3_	Teaching design M_31_	The course design embodies high-level, innovation and challenge. It has the characteristics of reasonable setting and teaching students according to their aptitude. Personalized teaching programs are designed according to time, environment, people and needs
	Pattern recognition M_32_	Teaching and learning modes conform to students’ cognitive rules and acceptance characteristics
	Teacher-student interaction M_33_	Enhancing communication and interaction between teachers and students and students in various forms inside and outside class
Course effectiveness M_4_	Goal attainment M_41_	The goal of the course is to help students to master the required technical knowledge and skills, with a degree of achievement that could be measured, to evaluate the students’ learning effect in the whole process, give feedback in time and clear, to stimulate the students’ inner motivation, and to mobilize them to take the initiative to participate in the study
	Student acquisition M_42_	Students will gain knowledge, strengthen their abilities, and build their characters
	Student satisfaction M_43_	The recognition and satisfaction of students toward teachers’ teaching and courses will be promoted

## Results analysis

According to the evaluation indicator system constructed previously and the evaluation of each expert on the evaluation indicator, the FAHP and entropy method were used to determine the weight of each evaluation indicator.

### Determining the weight of each indicator by the FAHP

The evaluation results were quantified with reference to the FAHP 0.1–0.9 nine-scale quantitative method, and Table 3 was constructed after processing the scores of experts on indicators at each criterion level.

**Table 3:** Score of the importance of each indicator in the first-class undergraduate course quality evaluation criterion layer.

Quality evaluation of first-class undergraduate courses	Course concept	Course resources	Course organization	Course effectiveness
Course concept	0.50	0.75	0.63	0.72
Course resources	0.25	0.50	0.35	0.33
Course organization	0.37	0.65	0.50	0.62
Course effectiveness	0.28	0.67	0.38	0.50

On this basis, the fuzzy judgment matrix of undergraduate course quality evaluation was obtained as follows: $A=\left[\begin{array}{cccc} 0.50 & 0.75 & 0.63 & 0.72\\ 0.25 & 0.50 & 0.35 & 0.33\\ 0.37 & 0.65 & 0.50 & 0.62\\ 0.28 & 0.67 & 0.38 & 0.50 \end{array}\right]$ .

According to specific calculation Eqs. (1) and (2), the matrix *A* was transformed into the fuzzy consistency matrix *R*: $R=\left[\begin{array}{cccc} 0.50 & 0.69 & 0.58 & 0.63\\ 0.31 & 0.50 & 0.38 & 0.43\\ 0.42 & 0.62 & 0.50 & 0.55\\ 0.37 & 0.57 & 0.45 & 0.50 \end{array}\right]$ .

According to parameter Eq. (3) and the FAHP weight Eq. (4), where *n* is the dimension of the matrix, the values of w were calculated as *w*_1_ = 0.32, *w*_2_ = 0.18, *w*_3_ = 0.27, and *w*_4_ = 0.23, respectively.

According to the aforementioned data collation and calculation process, the weight of each indicator of undergraduate course quality evaluation is shown in Table 4.

**Table 4:** Weight of each indicator of undergraduate course quality evaluation by FAHP.

Target layer	Criterion layer	Weight	Target layer	Weight	Synthetic weight
Undergraduate course quality	Course concept	0.32	Value guidance	0.31	0.0992
			Students centering	0.39	0.1248
			Continuous improvement	0.3	0.0960
	Course resource	0.18	System renewal	0.24	0.1920
			Fit of content	0.5	0.4000
			Interdisciplinary integration	0.26	0.2080
	Course organization	0.27	Teaching design	0.41	0.1107
			Pattern recognition	0.28	0.0756
			Teacher-student interaction	0.31	0.0837
	Course effectiveness	0.23	Goal attainment	0.35	0.0805
			Student acquisition	0.4	0.0920
			Student satisfaction	0.25	0.0575

### Determining the weight of each indicator by entropy method

According to the evaluation scores of each indicator by experts and entropy method calculation Eqs. (5)–(9), the calculation process values and weights were obtained, as shown in Table 5.

**Table 5:** Weight of each indicator of undergraduate course quality evaluation calculated by the entropy method.

Target layer	*E_j_*	*D_j_*	*e_j_*
Value guidance	0.9929	0.0071	0.0129
Students centering	0.9868	0.0132	0.0241
Continuous improvement	0.9606	0.0394	0.0718
System renewal	0.9512	0.0488	0.0889
Fit of content	0.9903	0.0097	0.0177
Interdisciplinary integration	0.9818	0.0182	0.0332
Teaching design	0.9837	0.0163	0.0297
Pattern recognition	0.9624	0.0376	0.0685
Teacher-student interaction	0.8638	0.1362	0.2482
Goal attainment	0.8092	0.1908	0.3477
Student acquisition	0.9846	0.0154	0.0281
Student satisfaction	0.9840	0.0160	0.0292

### Combined FAHP: Entropy method to determine the comprehensive weight of each indicator

The comprehensive weight of each indicator was calculated according to Eq. (10), and the results are shown in Table 6.

**Table 6:** Comprehensive weights of various indicators of undergraduate course quality evaluation.

Target layer	wj	ej	βj
Value guidance	0.0992	0.0129	0.0123
Students centering	0.1248	0.0241	0.0290
Continuous improvement	0.0960	0.0718	0.0664
System renewal	0.1920	0.0889	0.1646
Fit of content	0.4000	0.0177	0.0683
Interdisciplinary integration	0.2080	0.0332	0.0666
Teaching design	0.1107	0.0297	0.0317
Pattern recognition	0.0756	0.0685	0.0499
Teacher-student interaction	0.0837	0.2482	0.2003
Goal attainment	0.0805	0.3477	0.2698
Student acquisition	0.0920	0.0281	0.0249
Student satisfaction	0.0575	0.0292	0.0162

### Calculating the results of undergraduate course quality evaluation

In this study, the fuzzy comprehensive evaluation method was selected to calculate the evaluation results of undergraduate course quality, and the specific steps are as follows: Step 1: determine the fuzzy factor evaluation set M= $\left\{m_{1},m_{2},m_{3},\cdots ,m_{i}\right\}$ ; step 2: determine the weight set W= $\left\{w_{1},w_{2},w_{3},\ldots ,w_{n}\right\}$ according to the calculated weight values of each indicator; and step 3: determine the fuzzy comment set V. In this study, five-grade standards, {very good, good, average, poor, and very poor} were adopted as the dimensions of first-class undergraduate course quality evaluation, that is, *V* = {very good, good, average, poor, and very poor}, which, respectively, correspond to five value ranges; step 4: invite relevant personnel to score each indicator of undergraduate course quality evaluation, and combine the comment set *V* to count out the scoring statistical summary table of each factor, and calculate the membership matrix *X* of the evaluation indicator; step 5: calculate the evaluation value of undergraduate course quality according to Eqs. (11), (12).


(11) $$B_{k}=W_{k}•X_{k}=\left(w_{1},w_{2},w_{3},\ldots ,w_{m}\right)•\left[\begin{array}{cccc} x_{11} & x_{12} & x_{13} & x_{1n}\\ x_{21} & x_{22} & x_{23} & x_{2n}\\ \vdots & \vdots & \vdots & \vdots \\ x_{m1} & x_{m2} & x_{m3} & x_{mn} \end{array}\right]$$

(12) S=B•V

### Evaluation of the course of “Management”

In this section, based on the case study of the first-class undergraduate course “Management” in Anyang Normal University, the application of undergraduate course quality evaluation system in practice was illustrated. In this study, 94 accounting undergraduate students of grade 2021 were selected as subjects and were invited to evaluate the course of “Management.” According to the survey results, the membership degree of each indicator was sorted out, as shown in Table 7.

**Table 7:** Weight and membership degree of each indicator of quality evaluation of “Management” course.

Criterion layer/weight	Indicator layer/weight	Membership
Course concept 0.32	Value guidance 0.31	(0.2, 0.5, 0.3, 0, 0)
	Student centering 0.39	(0.3, 0.4, 0.3, 0, 0)
	Continuous improvement 0.30	(0.3, 0.5, 0.2, 0, 0)
Course resources 0.18	System renewal 0.24	(0, 0.3, 0.3, 0.2, 0.2)
	Fit of content 0.50	(0.4, 0.4, 0.2, 0, 0)
	Interdisciplinary integration 0.26	(0, 0.2, 0.5, 0.2, 0.1)
Course organization 0.27	Teaching design 0.41	(0.3, 0.4, 0.3, 0, 0)
	Pattern recognition 0.28	(0.2, 0.4, 0.4, 0, 0)
	Teacher-student interaction 0.31	(0.2, 0.4, 0.4, 0, 0)
Course effectiveness 0.23	Goal attainment 0.35	(0, 0.1, 0.5, 0.3, 0.1)
	Student acquisition 0.40	(0.3, 0.2, 0.2, 0.1, 0)
	Student satisfaction 0.25	(0.2, 0.3, 0.4, 0.1, 0)

According to Eqs. (11) and (12), the evaluation values of course concept, course resources, course organization, and course effectiveness were calculated as [4.0494.0573.8222.69] , respectively, and the overall quality evaluation of “Management” course can also be calculated as 3.574 accordingly. The calculation process is as follows:


$$\begin{array}{l} B=W•X=\left(\begin{array}{cccc} 0.11 & 0.30 & 0.28 & \end{array}0.31\right)\\ \;\;\;\;•\left[\begin{array}{ccccc} 0.288 & 0.473 & 0.239 & 0 & 0\\ 0.092 & 0.301 & 0.321 & 0.55 & 0.33\\ 0.211 & 0.400 & 0.389 & 0 & 0\\ 0.034 & 0.118 & 0.471 & 0.274 & 0.087 \end{array}\right]. \end{array}$$

By calculating, it can be obtained: $B=\left(\begin{array}{ccccc} 0.1289 & 0.29091 & 0.38004 & 0.24994 & 0.12597 \end{array}\right)$ .

Then


$$\begin{array}{l} S=B•V\\ \;\;=(\begin{array}{ccccc} 0.1289 & 0.29091 & 0.38004 & 0.24994 & 0.12597) \end{array}•\left[\begin{array}{c} 5\\ 4\\ 3\\ 2\\ 1 \end{array}\right]\\ \;\;=3.57411. \end{array}$$

Judging from the evaluation results, the overall evaluation value of the “Management” course is 3.574. Specifically, the evaluation value of the course concept was 4.049 and that of course resources was 4.057. All the two elements were rated as “very good,” and the evaluation value of course organization was 3.822; the corresponding evaluation grade was “good,” and that of course effectiveness was 2.69, and the corresponding evaluation grade is “general.” Thus, it is clear that the “Management” course has the highest evaluation in the course concept, while the corresponding course effectiveness has the lowest evaluation.

## Results and discussion

### Main conclusion

On the basis of the aforementioned research on the evaluation of the quality of the “Management” course, it can be extended to other related undergraduate courses, and efforts can be made to evaluate the four first-level indicators in the course evaluation indicator system based on the results of the evaluation, experience in improving the quality of the course, and effective strategies:

#### Changing teaching concept from knowledge instillation to ability development and wisdom awakening

In the quality evaluation of the “Management” course, the evaluation value of “course concept” is only lower than that of “course resources,” and the corresponding evaluation grade is “very good.” Learning of knowledge points in management courses is generally online. Offline classroom, taking exploratory learning and group task learning as main organizational forms, focuses on cultivating students’ ability to solve practical problems, as well as basic management qualities, such as communication ability, teamwork ability, and innovation ability. After each learning unit, practice links are designed according to the learning content and students’ learning situation; corresponding chapter exercises and assignments are arranged for them, so as to evaluate their performance in all links, and the evaluation results are included in the course scores. The course focuses on the evaluation of students’ autonomous learning ability and pays attention to process and comprehensive evaluation, including comprehensive evaluation of students’ participation in learning discussions, exercises, works submitted in practice, or practical activities organized in the teaching process. In the whole teaching process, teachers focus on guiding and supporting students’ autonomous learning, rather than just instilling knowledge. In this way, the students have completed the transformation from being overwhelmed at the initial stage to being confident in the middle and late stages, from being afraid of facing group tasks to being calm at the later stage, and from completing knowledge learning assigned by teachers to exercising and improving conscious self-management ability. The considerable change brought by one semester’s course study to students lies in applying management knowledge to the practice of self-management, which constantly brings the improvement of management ability and the awakening of management wisdom.

#### Changing from textbook resources to online + offline course platform resources

In the course quality evaluation of “Management,” the “course resources” has the highest evaluation value and the “very good” evaluation grade, which benefits from the continuous construction of management online resources in 10 years. At present, the online course resources of the “Management” course include self-made teaching videos, open class videos, courseware, cases, exercise libraries, homework libraries, classroom activities, and group tasks on platforms such as NetEase. All classes in the course use the “online+ offline” mixed teaching method. Online students are guided to learn autonomously by means of task publishing, assignment arrangement, task orientation, etc. Offline students are reinforced and improved by classroom activities, such as seminars and scene simulations. Then, a targeted improvement plan will be put forward and implemented in the following teaching activities to continuously improve the teaching effect and the quality of the course according to the students’ performance in the normal teaching process, including their understanding and mastery of knowledge points, their ability to analyze and solve practical problems by using relevant theoretical knowledge, and the improvement of management literacy, combined with the data and information of the course process management.

#### Transforming from teaching-centered to learning-centered teaching organization

The evaluation grade corresponding to the evaluation value of “course organization” in the course quality evaluation of “Management” is “good.” In the process of course organization, the “learning-centered” concept has always been adhered to. Through a careful teaching design, the course teaching team has fully stimulated the students’ learning enthusiasm and initiative, making them gradually become the main body of the classroom and the power source of the learning process. In the past two semesters, the “Management” course has adopted a mixed online and offline teaching mode. Only online interactive person-time can reach more than 13 times the number of students enrolled each semester, and the interactive rate of student–question–teacher–answer and student–question–answer reaches more than 50%. When the relationship between “teaching” and “learning” is straightened out in the course organization process, the interaction between teachers and students should be strengthened in the teaching design of teachers to create a learning situation in which teachers and students participate together. In the later stage, teachers should exit consciously and appropriately to build a student-oriented atmosphere of debate and discussion-type learning, so that students’ learning is more exploratory and personalized. When students’ learning demands and behaviors become active, their active thirst for knowledge and inquiry learning behaviors will become the driving force of the whole course quality improvement, which will promote the course learning state from teacher leading to teacher-asking–student-answering, to teacher–student dialog, and to student-asking–teacher-evaluating, and finally realize equal knowledge discussion and debate between teachers and students and between students, so as to truly realize the “learning”-centered course quality improvement.

#### Transforming teaching effect from “teaching reflection” to “teaching and learning reflection”

The evaluation value of “course effectiveness” of “Management” is the lowest among the four first-level indicators of course evaluation, and the corresponding evaluation grade is “general,” which needs special attention in the course construction process of it in future. The improvement and promotion of curriculum quality lies not only in the improvement of teachers’ teaching ability but also in the improvement of students’ learning ability. Therefore, the reflective subject in each stage should include not only teachers but also students. Teachers’ teaching reflection in the teaching of “Management” should be carried out in various forms, such as teaching observation and teaching discussion, in each class, chapter, mid-term, and semester by individual teachers and course teams. The evaluation criterion of the “good teaching effect” is no longer that the teaching materials are well-explained but that the teaching academic level is improved on the basis of discipline academic self-restraint. The ultimate effectiveness measurement criterion is “how well the students learn,” “how the students learn,” and so on, which is a great challenge for teachers. Previously, to achieve good teaching results, it took a good blacksmith to make steel. Now teachers are required to “not only impart high-quality knowledge to students but also motivate students to learn.” Once students have reached the stage of active learning, teachers should also ensure that they can provide sufficient and high-quality knowledge. Furthermore, with the continuous occurrence of scientific and technological revolution and industrial change, and the ever-increasing speed of knowledge updating, teachers are required to constantly learn in the process of promoting students’ active learning, paying close attention to the frontier and changes of disciplines all the time, not only focusing on the “discovered learning” but also improving the “comprehensive learning,” “applied learning,” and “teaching learning” (Cai et al., 2018), thus ensuring the improvement of curriculum quality with the improvement of their own academic accomplishment. At the same time, reflection on teaching and learning should be carried out among students in the form of study notes, group discussions, and class discussions; teachers’ teaching should be reflected from the perspective of active learning; and learning activities should be reflected from the perspective of self-management, so as to promote the mutual growth of teachers’ and students’ abilities through the two-way reflection on teaching and learning and then to realize the improvement of teaching and learning that benefits both teachers and students.

### Strengths and weaknesses of this study

In this study, the quantitative and qualitative evaluation methods are first combined to construct the evaluation indicator of the curriculum by literature research and expert evaluation, and then the fuzzy analytic hierarchy process and entropy method were used to evaluate the course quality. Finally, based on the results, measures for improving the course quality are proposed. The application of the quantitative and qualitative methods in the study on improving the course quality provides a new perspective for the study of education and teaching. However, the research methods still have some disadvantages, such as the complex process of the multi-time indicator test, which needs to be further improved in the related research in future.

### Significance and prospect of research

In China, the scale of undergraduate enrollment in 2021 was 4.446 million, and the improvement of curriculum quality is of practical significance to ensure the quality of undergraduate talent training. The basic focus of undergraduate education and teaching reform is curriculum reform, that is, through the evaluation of the course quality, schools, teachers, and students can be guided to participate in the process of course construction and organization and implementation, and the key indicators can be put into the process accordingly. Through the management cycle, the course quality can be continuously optimized and continuously improved, so as to achieve the comprehensive effect of improving the course quality, such as the goal of course construction, the multi-dimensional cultivation of students, the diversity of teaching platforms, the diversity of course resource allocation, the diversity of classroom activities, the autonomy of students’ learning, the multi-channel communication between teachers and students, the individuation of course guidance, and the flexibility of examination methods.

### Future research methods

The reform of undergraduate education is endless, and the improvement of undergraduate course quality is always on the way. Therefore, in future teaching and research work, how to design a new evaluation indicator system based on the new characteristics in practice, such as how to design the evaluation indicator with the combination of online and offline teaching methods, and how to use more effective methods to evaluate the quality of the curriculum and other issues should be the research focus.

## Data availability statement

The original contributions presented in the study are included in the article/supplementary material, further inquiries can be directed to the corresponding author.

## Ethics statement

Ethical review and approval was not required for the study on human participants in accordance with the local legislation and institutional requirements. Written informed consent from the patients/ participants was not required to participate in this study in accordance with the national legislation and the institutional requirements

## Author contributions

AP contributed to conception and design of the study, organized the database, performed the statistical analysis, and wrote the first draft of the manuscript.

## Funding

This paper was financially supported by Training Program for Young Core Teachers in Higher Institutions of Henan Province *Research on the Quality Promotion Mechanism of First-class Undergraduate Course from the Perspective of PDCA* (no.: 2021GGJS128) and served as staged outcomes of the Research Program of Teacher Education Course Reform in Henan Province *Construction of the Core Quality System of Wushu Teachers in Primary and Middle Schools under the Background of Building a Powerful Sports Country* (no.: 2021-JSJYYB-121) and The 14th Five-Year Planning Subject of Educational Science in Henan Province *Relationship between Disciplines and Major Construction in Local Colleges and Universities* (no.: 2021YB0193).

## Conflict of interest

The author declares that the research was conducted in the absence of any commercial or financial relationships that could be construed as a potential conflict of interest.

## Publisher’s note

All claims expressed in this article are solely those of the authors and do not necessarily represent those of their affiliated organizations, or those of the publisher, the editors and the reviewers. Any product that may be evaluated in this article, or claim that may be made by its manufacturer, is not guaranteed or endorsed by the publisher.
